# N-Acetylcysteine Administration Attenuates Sensorimotor Impairments Following Neonatal Hypoxic-Ischemic Brain Injury in Rats

**DOI:** 10.3390/ijms232416175

**Published:** 2022-12-19

**Authors:** Evangelia Kesidou, Christina Bitsina, Athanasios Chatzisotiriou, Paschalis Theotokis, Evgenia Dandi, Despina A. Tata, Evangelia Spandou

**Affiliations:** 1 Laboratory of Experimental Neurology and Neuroimmunology, 2nd Department of Neurology, AHEPA University Hospital, Aristotle University of Thessaloniki, 546 36 Thessaloniki, Greece; 2 Laboratory of Physiology, Faculty of Medicine, Aristotle University of Thessaloniki, 541 24 Thessaloniki, Greece; 3 Laboratory of Cognitive Neuroscience, School of Psychology, Aristotle University of Thessaloniki, 541 24 Thessaloniki, Greece

**Keywords:** hypoxia, ischemia, brain injury, N-acetylcysteine (NAC), inducible nitric oxide synthase (iNOS), sensorimotor tests, neuroprotection

## Abstract

Hypoxic ischemic (HI) brain injury that occurs during neonatal period has been correlated with severe neuronal damage, behavioral deficits and infant mortality. Previous evidence indicates that N-acetylcysteine (NAC), a compound with antioxidant action, exerts a potential neuroprotective effect in various neurological disorders including injury induced by brain ischemia. The aim of the present study was to investigate the role of NAC as a potential therapeutic agent in a rat model of neonatal HI brain injury and explore its long-term behavioral effects. To this end, NAC (50 mg/kg/dose, i.p.) was administered prior to and instantly after HI, in order to evaluate hippocampal and cerebral cortex damage as well as long-term functional outcome. Immunohistochemistry was used to detect inducible nitric oxide synthase (iNOS) expression. The results revealed that NAC significantly alleviated sensorimotor deficits and this effect was maintained up to adulthood. These improvements in functional outcome were associated with a significant decrease in the severity of brain damage. Moreover, NAC decreased the short-term expression of iNOS, a finding implying that iNOS activity may be suppressed and that through this action NAC may exert its therapeutic action against neonatal HI brain injury.

## 1. Introduction

Hypoxic-ischemic (HI) brain injury during the perinatal period is a major cause of lifelong neurological disability and mortality. A large proportion of infants who suffered a perinatal HI insult are at high risk of experiencing developmental delays, sensorimotor deficits, and cognitive impairments [1,2]. Although advances in technology and medicine ensure early diagnosis and therapeutic intervention culminating in increased survival rates, it is imperative for researchers to target multiple mediators which are involved in unexplored complex pathways of brain injury [3]. Drawing attention to novel and efficacious therapies will be crucial to tackle long-term sensorimotor and cognitive impairments that persist into adulthood [4].

The onset of HI injury is characterized by oxygen deprivation and the interruption of blood flow to the infant’s brain which subsequently trigger a cascade of biochemical and molecular mechanisms eventually leading to permanent brain damage. Main features of the pathophysiology of HI-related brain damage include excitotoxicity, accumulation of intracellular calcium, formation of reactive oxygen species (ROS), inflammation and necrotic, apoptotic or autophagic cell death [5,6,7]. HI brain injury is a complex evolving process characterized by an early phase of primary energy failure followed by secondary energy failure defined by oxidative dysregulated metabolism that can last for days or even weeks after the insult [8,9,10].

Numerous studies have explored the efficacy of potential neuroprotective strategies including growth factors (e.g., erythropoietin, G-CSF), antioxidants, magnesium sulfate, and stem cells in various animal models of neonatal HI [11,12,13]. Currently, therapeutic hypothermia is the only standard treatment used in newborns suffering from HI, although it is not consistently efficient in severe HI encephalopathy [14,15,16]. Therefore, the development of novel neuroprotective therapeutic approaches is crucial for neonates with HI encephalopathy.

N-acetylcysteine (NAC) is a precursor of glutathione (GSH) and is a possible neuroprotective agent considering that it crosses the blood brain barrier [17]. Based on existing evidence, NAC alleviates oxidative stress and offers neuroprotection in animal models and human studies in a variety of neurological disorders mainly through its actions as a free radical scavenger, stimulating GSH synthesis, and promoter of glutathione transferase activity [18,19,20,21,22,23,24]. NAC also regulates the NF-kB-dependent production of proinflammatory cytokines and inhibits inducible nitric oxide synthase (iNOS) and TNF-α expression, thus acting as an important modulator of inflammatory response induced by neonatal HI [25,26,27].

In neonatal models of HI, but also in the combined HI/inflammation model, NAC administration alone or in therapeutic combination with hypothermia and/or vitamin D has proved effective in reducing infarct volume, preventing demyelination and improving early and late functional deficits [28,29,30]. According to recent magnetic resonance spectroscopy (MRS) data, administration of NAC to human neonates with HI encephalopathy reduces ROS and subsequently oxidative stress via increase in GSH levels [31]. However, there is a relative lack of studies regarding long-term behavioral and histopathological alterations following NAC administration as monotherapy in animal models of neonatal HI brain injury. To the best of our knowledge, there is only one similar study reporting the beneficial effect of NAC on long-term motor function on HI animals by attenuating white matter injury [32], but its effect still remains unclear. In the present study, we aimed at exploring the possible neuroprotective effect of NAC in a preclinical model of neonatal HI. To this end, we investigated whether NAC administered prior to and following HI may have a beneficial effect on brain damage and long-term sensorimotor deficits. Moreover, we performed additional sensorimotor tests to confirm the attenuation of sensorimotor functions with NAC single-agent administration. In parallel, we implemented immunohistochemistry assay for iNOS expression, a potential target of NAC, and strong indication of its decrease was noted. Thus, our findings support the suggestion that NAC could be a promising candidate for the treatment of HI injury and resulting disorders.

## 2. Results

### 2.1. Effect of NAC on Sensorimotor Function 

The possible neuroprotective action of NAC in long-term functional outcomes of HI damage was assessed by the following five sensorimotor tests (a) the grip traction test, (b) the foot fault test, (c) the postural reflex test, (d) the limb-placing test with various sensory stimuli, and (e) the accelerated cylinder (Rota-Rod). The tests were performed for 3 days between PND42 and PND46.

#### 2.1.1. Grip Traction Test

**Figure 1:**
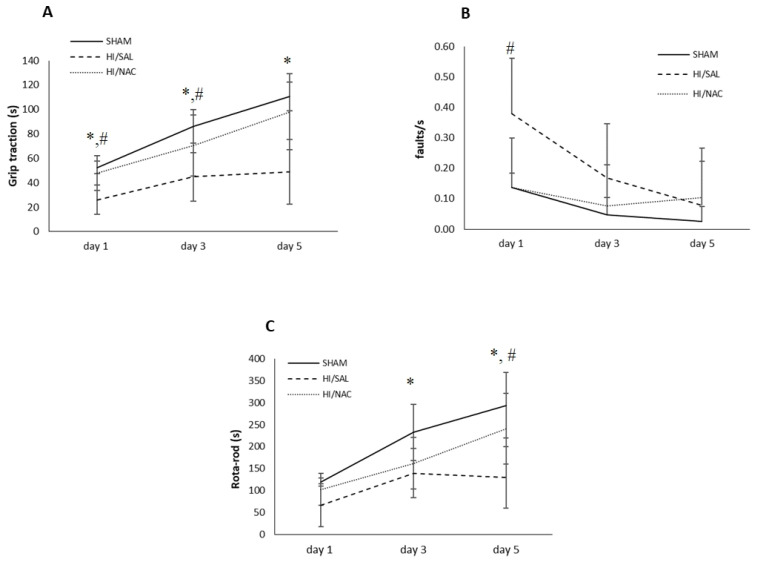
(**A**) Grip traction, (**B**) Foot-fault and (**C**) Rota-Rod test of SHAM-operated, HI/SAL and HI/NAC treated animals during the 3 days of observation period. Data are presented as mean ± SD. (*) *p* < 0.05 HI/SAL vs. SHAM group, (#) HI/SAL vs. HI/NAC group.

There was an improvement in the performance of the grip traction test (Figure 1A) among all the three groups during the 3 days of experiment, as shown by the strong main effect of days in the repeated-measures ANOVA [F(2, 38) = 55.385, *p* < 0.01, *η*^2^ = 0.745]. There was also a significant main effect for group [F(2, 19) = 10.634, *p* < 0.01, *η*^2^ = 0.528].

A significant interaction was shown between group and timing [F(4, 38) = 4.453, *p* < 0.01, *η*^2^ = 0.319), showing that the rate of improvement was not the same for all groups of animals. Specifically, in the SHAM operation group and the operated HI/SAL control group, improvement took place between days 1 and 3, while in the HI/NAC group, animals improved significantly from day 3 to day 5 as well. At the last day of the experiment, values were 110.50 ± 11.79, 48.91 ± 30.872 and 98.14 ± 26.492 for SHAM/HI, SAL/HI and NAC animals, respectively.

We then proceeded with the comparison between groups per separate day. All days disclosed significant differences. Post-hoc Bonferroni tests showed that SHAM animals significantly differed from HI/SAL animals at all time points. HI/NAC animals did not differ from SHAM, while they differed from HI/SAL, although in day 3 their difference did not reach statistical significance.

#### 2.1.2. Foot Fault Test 

An initial placing deficit during locomotion on the grid was shown by all groups of rats (Figure 1B). A significant effect was shown for days [F(2, 38) = 5.759, *p* < 0.05, *η*^2^ = 0.233)], as well as for groups [F(2, 19) = 4.098, *p* < 0.05, *η*^2^ = 0.301]. The interaction between group and days was not shown to be significant [F(4, 38) = 2.072, *p* > 0.05]. Post Hoc Bonferroni tests showed that SHAM and HI/NAC animals improved, although not significantly, while HI/SAL improved, although not significantly, while the HI/SAL improved significantly from day 1 to day 3. The HI/SAL animals differed significantly from HI/NAC only on day 1.

#### 2.1.3. Rota-Rod Test

Daily performance of all groups is presented in Figure 1C. Animals of all three groups improved in their performance over the 3 days of testing as shown by a repeated-measures ANOVA, which indicated a significant main effect for days [F(2, 38) = 39.62, *p* < 0.01, *η*^2^ = 0.68] and for group [F(2, 19) = 7.865, *p* < 0.01, *η^2^* = 0.453].

There was a significant interaction effect between group and timing [F(4, 38) = 3.997, *p* < 0.01, *η*^2^ = 0.3], showing that groups had different rates of improvement. SHAM and HI/SAL groups did not differ significantly from day 3 and day 5, while in the HI/NAC group improvement was constant and all days differed significantly from each other (*p* < 0.05).

#### 2.1.4. Postural Reflex Test

**Figure 2:**
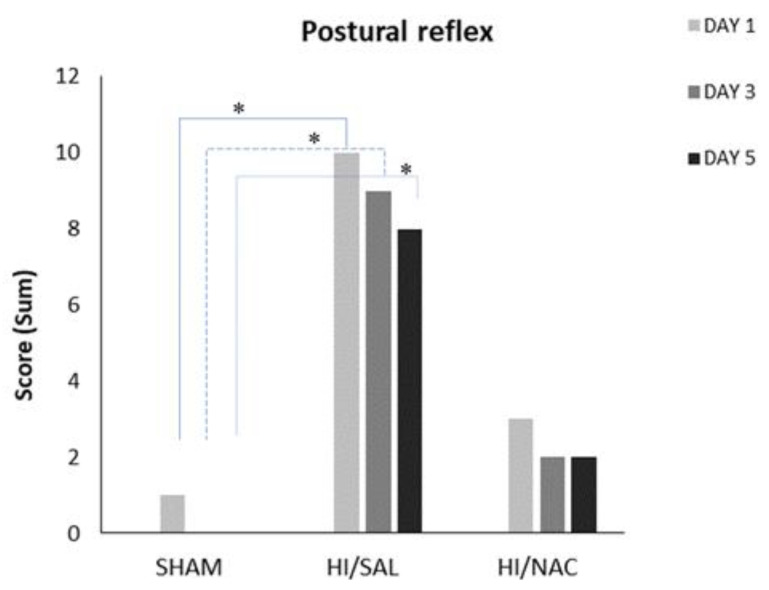
Performance of SHAM, HI/SAL and HI/NAC groups during the 3 days of examination. (*) *p* < 0.05 HI/SAL vs. SHAM group.

The postural reflex test showed that cerebral HI seriously affects the sensorimotor function of the animals (Figure 2). Cochran’s Q test was separately applied to each group and did not show significant differences, as the percentage of animals with abnormal reflex remained fairly constant during all days. On the first day 3/4, 1/11 and 4/7 (SHAM/HI, SAL/HI, NAC animals, respectively) had normal reflex, while on day 5 the proportion was 4/4, 3/11 and 5/7. Chi-square test showed that HI/SAL animals differed significantly from SHAM, while the difference from HI/NAC was not significant. 

#### 2.1.5. Limb Placing Tests

Generally, the majority of SHAM and HI/NAC animals responded correctly in all limb placing tests, while HI/SAL treated animals exhibited a relatively poor performance. 

**Figure 3:**
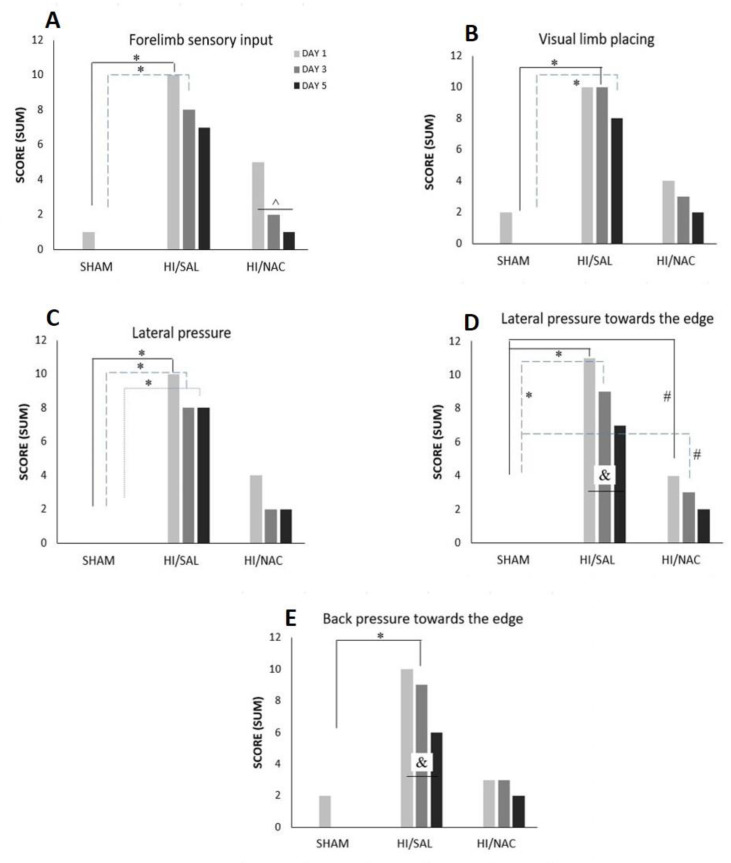
(**A**–**E**) Performance of SHAM, HI/SAL and HI/NAC groups in limb placing tests during the 3 days of the examination. (*) *p* < 0.05 HI/SAL vs. SHAM group, (#) *p* < 0.05 HI/NAC vs. SHAM group, (&) *p* < 0.05 HI/SAL day1 vs. day5, (^) *p* < 0.05 HI/NAC day1 vs. day5.

Forelimb sensory input: SHAM animals presented with (3/4) on day1 and (4/4) on day 5 (4/4), while HI/SAL animals improved from 1/11 to 4/11, albeit not significantly (*p* > 0.05). HI/NAC showed significant improvement from 2/7 on day1 to 6/7 in day 5 (Cochran’s Q test, *p* < 0.05). On day 1 and day 3 the SHAM animals differed significantly from HI/SAL (Chi-square, *p* < 0.038 and 0.027 respectively), while HI/NAC did not differ either with SHAM or with HI/SAL. On day 5 the difference between groups was not significant (Figure 3A).

Visual limb placing: SHAM animals improved from day 1 (2/4) to day 5 (4/4), HI/SAL improved from 1/11 to 3/7 and HI/NAC animals from 3/7 to 5/7, although none of these differences reached statistical significance (Cochran’s Q test, *p* > 0.05). There was no significant difference between groups on day 1 (Chi squared, *p* > 0.05), but on day 3 and day 5, SHAM animals clearly differed from HI/SAL (*p* < 0.01 and *p* < 0.05 respectively), while HI/NAC did not differ either from SHAM or from HI/SAL (Figure 3B).

Lateral pressure: SHAM animals presented with normal reflex from day 1 (4/4), while HI/NAC animals improved from 3/7 in day 1 to 5/7 in day 5, although this difference did not reach statistical significance (Cochran’s Q test, *p* > 0.05). HI animals improved from 1/11 correct tests in day 1 to 3/11 in day 5, but this difference was not significant (Cochran’s Q test, *p* > 0.05). Pairwise comparisons for each day showed that SHAM animals significantly differed from HI/SAL (Chi-square, *p* < 0.05), while the difference between HI/NAC and either SHAM or HI/SAL did not reach statistical significance (Figure 3C).

Lateral pressure towards the edge: SHAM animals presented with normal reflex from day 1 (4/4), while HI/NAC animals improved from 3/7 in day 1 to 5/7 in day 5, although this difference did not reach statistical significance (Cochran’s Q test, *p* > 0.05). HI/SAL animals significantly improved from 0/11 correct tests in day 1 to 4/11 in day 5 (Cochran’s Q test, *p* = 0.05). Comparisons per day showed that HI/SAL animals differed significantly from the other groups on day 1 and day 3 (Figure 3D).

Backpressure towards the edge: SHAM and HI/NAC animals did not show any significant improvement in their performance (Cochran’s Q test, *p* > 0.05), while HI/SAL animals gradually improved from 1/11 correct tests in day 1 to 5/11 in day 5 (Cochran’s Q test, *p* > 0.05). HI/SAL animals scored significantly less than SHAM animals on day 3 (Chi Square, *p* = 0.01) (Figure 3E).

### 2.2. Neuroprotective Effect of NAC on HI-Induced Brain Damage

**Figure 4:**
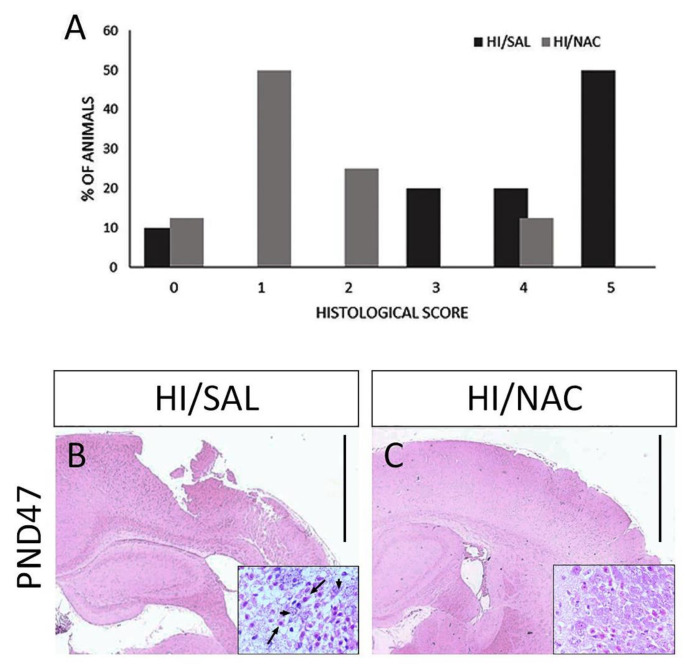
Effect of neonatal HI and NAC administration on brain damage. (**A**) Percentage of animals according to various degrees of brain damage based on a 6-point scale. Representative coronal brain sections of HI/SAL and HI/NAC-treated rats at 7 weeks following neonatal HI under H&E staining. Extensive damage of the ipsilateral cerebral cortex is observed in HI/SAL group (**B**). In HI/NAC group, brain damage is significantly limited and cerebral cortex is preserved (**C**). Scale=100 μm. CA1 region of hippocampus is shown under higher magnification ×40. Black arrows indicate damaged neurons with eosinophilic cytoplasm and pyknotic nucleus. Arrowheads indicate neurons with relatively normal morphology.

Following the completion of the sensorimotor testing, the rat brains were microscopically examined and classified according to the degree of damage of the cerebral cortex and the hippocampus. Histopathological analysis confirmed the presence of severe brain damage induced by HI. There was infarction involving almost all regions of the hemisphere that underwent carotid ligation, although it was more pronounced in the middle cerebral artery region (brain damage > 50%, score 4 and 5) in 70% of HI/SAL treated rats. A significant reduction in the extension of brain injury was observed in the ipsilateral hemisphere of HI/NAC treated rats compared to the HI/SAL group (*p* < 0.05) (Figure 4A,B). In 60% of HI/NAC treated rats, the extension of brain damage was less than 25% (score 1 and 2) with the hippocampus and cerebral cortex being preserved or exhibiting sporadic areas of damaged neurons (Figure 4A,C). Similar histopathological changes were observed in short-term study. In the ipsilateral hemisphere of 74% of HI/SAL animals there was a significant tissue injury (brain damage > 50%, score 4 and 5). On the contrary, mild brain damage (<25%, score 1 and 2) was observed in 55% of HI/NAC animals. 

### 2.3. Effect of HI and NAC Administration on iNOS Expression

**Figure 5:**
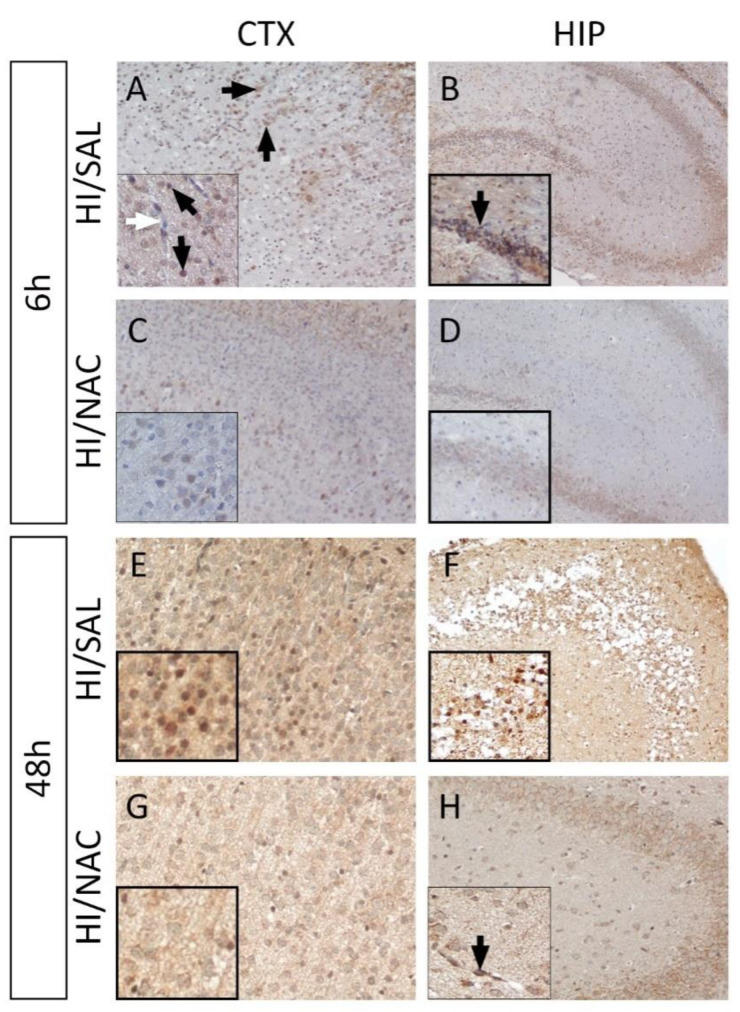
NAC administration reduces iNOS expression in NAC/HI group. Representative immunohistochemical staining for iNOS expression (DAB-brown staining) in the cortex-CTX (**A**,**C**,**E**,**G**) and hippocampus-HIP (**B**,**D**,**F**,**H**) from each group at 6 h and 48 h following neonatal HI. Lower levels of iNOS expression are detected both in CTX and HIP in NAC/HI group compared to the control (HI/SAL). Regions with extensive damage appeared to have diminished iNOS expression due to neuronal loss. Blackarrows indicate the positive cells and vessels and the white arrow denotes the absence of staining in bloodvessels (Magnification = ×40).

Elevated immunoreactivity of iNOS was observed 6 h after the end of the hypoxic-ischemic episode both in the cerebral cortex and the hippocampus of the HI/SAL group. Decreased iNOS expression was detected in regions with severe damage in the cortex (in the lesion core), which could be attributed to neuronal loss. In the peripheral region of the lesion, expression of iNOS was delineated in nerve cells and cells with morphological features of glial cells. In the hippocampus, a positive iNOS staining of different intensity was depicted mainly in the pyramidal cells of the CA1 and CA3 regions. In contrast to nerve cells, there was no particularly high expression of iNOS in blood vessels (Figure 5A). A similar pattern of iNOS expression with particularly increased intensity was observed at 48 h after the end of the HI episode. Regarding cells with glial morphology located within the lesion (core and peripheral area), positive staining was not observed, whereas iNOS appeared to have strong immunoreactivity with vessels in the cortex and hippocampal region (Figure 5B).

In contrast, in the HI/NAC group low levels of iNOS expression were reported in the cerebral cortex and especially in neuronal cells. Decreased expression of iNOS was also observed in the hippocampus although some vessels were noticed to be positively stained (Figure 5C,D and Figure S1).

## 3. Discussion

It is known that oxidative stress and inflammation are closely linked and involved in the pathogenesis of HI brain injury. The brain, and in particular the immature brain, is selectively vulnerable to oxidative stress due to its high oxygen consumption, high levels of polyunsaturated fatty acids, and low levels of antioxidant molecules [33,34,35]. Therefore, antioxidant agents that prevent or limit free radical production are considered as potentially effective therapeutic interventions for neonatal HI brain injury [36].

NAC is a precursor of L-cysteine that prevents GSH depletion and acts as a scavenger of free radicals [37]. Over the decades, clinical application of NAC has been linked to a broad range of conditions including acetaminophen toxicity and pulmonary oxygen toxicity. Recent findings from animal and human studies strongly support the beneficial role of NAC in a variety of neurological disorders, including neurodegenerative and vascular diseases [21].

The present study aimed at investigating whether NAC administered, at a relatively low dose, before and after the induction of HI in neonatal rats prevents brain damage and improves long-term behavioral impairments. In addition, we assessed iNOS expression in order to investigate whether the suggested neuroprotective effect of NAC is mediated by inhibiting NO production. 

The assessment of the long-term effect of NAC on motor coordination, muscle strength and limb placing reflexes of adult rats subjected to HI insult during the neonatal period was performed using five different sensorimotor tests. It is well established in the literature that the Rice–Vannucci model of neonatal HI produces extensive brain tissue damage sustained until adulthood [38,39]. Despite the fact that adult animals subjected to HI during the neonatal period appear essentially normal without displaying obvious motor deficits, locomotor abnormalities can be observed during neurological function assessment [40,41,42]. In line with this, in the present study, neonatal HI resulted in significant impairment in sensorimotor function at 42 days of age. In almost all individual tests, the performance of HI/SAL animals was significantly impaired compared to the performance of the control animals. Our findings are in accordance with previous studies reporting that sensorimotor function evaluated by grip traction, foot-fault tests as well as postural reflex and limb placing tests is impaired approximately at 5–6 weeks after neonatal HI [43]. 

Furthermore, motor coordination, assessed by Rota-Rod test, has been shown to be severely affected up to 9 weeks following the neonatal HI episode. However, there has been some controversy regarding the effect of neonatal brain injury on motor coordination. Previous reports demonstrate that motor coordination is not affected significantly by HI [44,45]. These contradictory findings may be due to differences in duration of hypoxia, ligated site of carotid artery, age of the animals, but most importantly may be attributed to differences in Rota-Rod protocol. Examination for a longer period of time and at an increasing rotating speed, as applied in our study, might be needed in order to reveal long lasting motor deficits caused by HI during the neonatal period.

In parallel, NAC was able to improve brain recovery in terms of sensorimotor function. NAC administration significantly improved the performance in most of the behavioral tests. In some cases, the performance of HI/NAC animals was similar to that of control animals (Rota-Rod, grip traction tests, postural reflex and part of limb placing tests). To the best of our knowledge, there are only limited data concerning the beneficial effect of NAC administered as a monotherapy against long-term impairments induced by neonatal HI. Our finding is in accordance with a previous study showing that low-dose (100 mg/kg) long-term NAC administration after neonatal HI resulted in motor dysfunction recovery in adult animals as evaluated by the Rota-Rod test. Long-term administration of NAC in combination with hypothermia has been shown to provide a significant improvement on neuromotor outcomes following severe neonatal HI compared to hypothermia treatment alone [28]. Furthermore, NAC in a therapeutic combination with vitamin D improved long-term cognitive and motor function over hypothermia alone in a neonatal rat model of severe HI [30].

The beneficial impact of NAC on functional outcome was accompanied by a significant reduction of the severity of brain damage at 7 weeks of age. In line with existing evidence, neonatal HI caused severe brain damage in 70% of the animals involving all the areas of the ipsilateral hemisphere that are more vulnerable to HI (i.e., cerebral cortex, hippocampus, striatum). NAC administration resulted in a marked preservation of brain tissue loss with almost 60% of the animals exhibiting only mild lesions. Taking into account the important role of the sensorimotor cortex in the network mediating the locomotor activity, the attenuation of brain damage in the cerebral cortex may imply an association between NAC neuroprotective action and restoration of sensorimotor function in HI animals. 

Previous studies have also reported the efficacy of NAC on reducing brain injury produced by neonatal HI and/or inflammation, although the administration protocol was different, using a higher dose (200 mg/kg) or a long-term treatment [16,46]. In addition, several studies have demonstrated the neuroprotective role of NAC in a combined therapeutic strategy with vitamin D and/or hypothermia [28,29,30]. It is worth mentioning that the recommended effective dose of NAC is at 50–200 mg/kg used as a single or repeated administration. Our preliminary experiments revealed that the optimum neuroprotective dose was 50 mg/kg/dose, whereas a high dose of NAC (500 mg/kg/dose, i.p.) administered prior to and following HI resulted in exaggerated HI-associated brain damage. This finding is in accordance with previous studies reporting a toxic dose-dependent effect of NAC [28,47,48], probably associated with its ability to increase both reduced and oxidized glutathione causing reductive stress. The aforementioned preclinical data are supported by a recent clinical study in neonatal HI encephalopathy reporting that a low dose of NAC (25–40 mg/kg/dose) co-administered with active vitamin D was well tolerated without serious adverse events and resulted in decreased oxidative stress in plasma and central nervous system as well as in favorable developmental outcomes [49].

According to existing research, the outcome of neonatal HI seems to be mediated by sex. Several mechanisms such as inflammatory response, oxidative stress and cell death pathways are recognized to play a key role in sex-related differences observed in neonatal HI brain injury [50,51,52,53,54]. This evidence underlines the importance of exploring sex-specific effects of therapeutic interventions. It should be noted that in the present study a possible sex-related effect of NAC has not been examined because the number of animals in sex-related subgroups was too small to detect differences. Previous pre-clinical studies reported that only HI female rats benefitted from a short-term combined treatment of NAC and hypothermia, indicating a sex-related effect of oxidative stress [28]. However, the underlying mechanism of this difference is not fully elucidated, and additional experiments need to be undertaken.

Previous studies have mentioned that NAC exerts its anti-oxidant, anti-inflammatory and anti-apoptotic actions through multiple mechanisms such as suppression of TNF-α, as well as inhibition of caspase-3 activity [25,26] and of NO production [28]. Altered NO synthesis and iNOS induction during the reperfusion phase have been implicated at least in part in the pathophysiological changes of ischemia/reperfusion injury in neonatal and adult animal models [55,56,57]. Upregulation of iNOS is triggered by several stimuli, including proinflammatory mediators, and transcriptional factors (e.g., NF-kB or HIF-1a) which are activated during brain ischemia [58,59]. Subsequently, induction of iNOS under redox state dysregulation and inflammatory conditions seems to play a key role in HI brain injury by leading to the production of large amounts of NO for longer periods [60,61]. Elevated levels of NO are involved in many pathophysiological pathways such as excitotoxicity, apoptosis and inflammation resulting in increased brain lesions [62]. Various therapeutic agents reduce the infarct volume and exert their protective role through the inhibition of iNOS expression. In addition, mice lacking iNOS gene are less vulnerable to HI brain injury suggesting that iNOS inhibition might be a potential neuroprotective strategy in HI brain injury [61,63]. NAC inhibits lipopolysaccharide-induced iNOS expression and NO production [64] in mature and developing rat brain [65]. NAC also efficiently blocks the induction of iNOS, thus preventing NO-induced toxicity in adult models of brain ischemia [20,27,47]. To the best of our knowledge, there is only one study reporting that combined treatment of NAC and hypothermia decreased iNOS expression in a neonatal rat model of HI [28]. This finding is in agreement with our results reporting decreased short-term iNOS expression following NAC administration. Although the difference in the intensity of the immunohistochemical staining clearly indicates the downregulation of iNOS expression following NAC treatment, the insufficient statistical power due to the small size of each group was a limitation to performing statistical analysis of the semiquantitative data. Suppression of iNOS activation and subsequent decreased NO production could reduce peroxynitrite-mediated formation of free radicals and inhibit apoptotic pathways and inflammatory response activated by HI. 

In conclusion, the present study demonstrated that NAC decreases brain damage and alleviates the long-term impairments in sensorimotor function in adult rats that were exposed to HI as neonates. In terms of mechanism, our findings indicate that the beneficial effect of NAC might be attributed to the suppressive action on iNOS expression. Improvement of long-term functional and histological outcomes indicate that NAC may also activate brain repair and plasticity mechanisms. Our findings suggest that NAC, a compound possessing anti-oxidative, anti-inflammatory and anti-apoptotic properties, may be a potential therapeutic intervention for the treatment of neonatal HI. Therefore, further studies are needed to confirm the safety profile of NAC, and explore the molecular mechanisms involved in its neuroprotective action as well as the optimal therapeutic window for neonatal HI brain injury. 

## 4. Materials and Methods

### 4.1. Animals

Female Wistar rats on the second gestational week were individually housed in standard laboratory cages until delivery. The day of birth was designated as postnatal day 0 (PND0). A total number of 72 pups of either sex were included in the experiment. Neonates were exposed to HI conditions on PND7 and then remained with their dams until weaning (PND23) on standard laboratory conditions (temperature: 22 ± 2 °C, 12:12 light/dark cycle: 08:00–20:00). Following weaning (PND 23), animals were housed in same-sex groups of two or three in standard laboratory cages, until the end of the experiment, with food and water *ad libitum*. All experimental procedures were conducted according to the institutional guidelines, in compliance with the Greek Regulations and the European Communities Council Directive (2010/63/EU) on the protection of animals used for scientific purposes. Experimentation received approval from the local Veterinary Medicines Directorate (# 471643-1811).

### 4.2. Neonatal Cerebral HI and Drug Administration

On PND7, pups were exposed to HI conditions as described by Rice et al. [38,66]. Briefly, the left common carotid artery was exposed through a midline neck incision, ligated with 5–0 silk suture and cut between the ligatures. After surgery, the rat pups were returned to their dams for 1.5–2 h recovery. In this model, neither the permanent unilateral carotid occlusion nor the hypoxia alone results in brain injury, but the combination of both causes moderate to severe brain damage in the ipsilateral to the ligation hemisphere [67,68,69]. Hypoxia was induced by placing the animals in a chamber, submerged in a 37 °C water bath and subjected to a humidified mixture of 8% oxygen-92% nitrogen for 1 h. Following hypoxic exposure, the animals were returned to their dams and allowed to recover until further experimentation. Pups from each litter were randomly assigned to 3 experimental groups: (a) Sham-operated rats (SHAM n = 15) subjected to the surgical procedure, but without undergoing artery ligation or being exposed to a hypoxic environment; (b) animals subjected to HI and treated before and immediately after the hypoxia with NAC (HI/NAC, 50 mg/kg each dose, ip, n = 27); animals subjected to HI and treated with similar volume by body weight of 0.9% saline (HI/SAL, n = 30). The administration protocol was based on previous reports utilizing NAC in neonatal rats and our preliminary experiments evaluating the efficacy of different doses of NAC on short-term histological outcomes [27,29]. In each experimental group, both male and female rats were combined because the number of animals in sex-related subgroups was too small to detect differences.

### 4.3. Sensorimotor Tests

At 42 days of age, sensorimotor function was evaluated. During the last five days before testing (PND37) the rats were handled by an experimenter blind to the group assignment. All tests were performed every other day, for a total duration of 3 days, beginning on PND42, and in the same order for all rats.

In the grip traction test [43,70], the muscle strength of the rat was tested by its ability to hang on a rope (0.5 cm diameter) placed horizontally 45 cm above the table by its forepaws. The time during which the rats remained (maximum 60 s) hanging on the horizontal rope was estimated.

In the foot fault test [43,71], the sensorimotor coordination of each rat was tested by placing the animal on a horizontal grid floor (50 × 40 × 40 cm, square size 3 × 3 cm, wire diameter 0.4 cm). A foot fault was defined as when the animal inaccurately placed a fore- or hind- limb and the paw fell through an opening in the grid floor. The number of foot-faults with regard to the time of movement during the 2 min of observation period was recorded.

Motor coordination and activity test using a Rota-Rod treadmill [41]. The rotating treadmill was set in motion at an accelerated speed of 4–40 rpm/min. Following the animal’s placement on the treadmill, the individual timers were started for a maximum of 10 min. If the animal fell off the treadmill the timer would stop.

In the postural reflex [43,72] assessment of forelimbs’ position was evaluated while the rat was held by the tail 50 cm above the table for 10–15 s. Normal rats extend both forelimbs towards the table (score 0). Rats with brain damage flex the forelimb contralateral to the damaged cerebral hemisphere (score 1).

In the limb-placing test [43,73], the experimenter held the rat, and the forelimb placement after different sensory stimuli was estimated as previously described in the published article [74]. Briefly, (1) forelimb sensory input was tested with the rat’s forelimbs touching a table edge. Normal score 0 corresponds to correct paw placing and score 1 to delayed and/or no placing. (2) Visual limb placing was assessed by lowering the rat toward a table. Normal score 0 refers to normal rats stretch and placement of both forepaws on the table whereas injured rats exhibit delayed and/or incomplete placing of the forelimb ipsilateral to the damaged hemisphere (score 1). (3) Rat placing onto the table and lateral force applied behind the shoulder until the forelimbs slid. Score 0 indicates normal rats’ resistance against the lateral pressure and score 1 corresponds to injured rats exhibiting reduced or no resistance against the lateral force. (4) Forelimb placement was evaluated by placing the rat on the table and lateral force was applied in order for the rat to approach the edge of the table. Score 0 shows the ability to grip onto the edge, whereas score 1 correlates with the inability of the injured rats to drop the forelimb contralateral to the injured hemisphere. Lastly, (5) the forelimb placement was tested as described above in (4), but a backpressure was applied.

### 4.4. Histological Analysis

Histological analysis was conducted for PND 10 (SHAM n = 5, HI/SAL n = 8, HI/NAC n = 8) and following the completion of sensorimotor testing (PND47, SHAM n = 10, HI/SAL n = 16, HI/NAC n = 13). Following the completion of sensorimotor testing (PND47), the animals were transcardially perfused under deep anesthesia with normal saline followed by 4% phosphate buffered paraformaldehyde solution. Subsequently, their brains were removed and embedded in paraffin. Serial sections at the level of the dorsal hippocampus (−3.24 mm to −3.36 mm posterior to bregma) were obtained and stained with hematoxylin-eosin. The degree of brain damage in the ipsilateral to the ligated carotid artery cerebral cortex and hippocampus was scored on a 6-point scale as follows:0 = normal, 1 = few neurons damaged (1–5%), 2 = several neurons damaged (6–25%), 3 = moderate number of neurons damaged (26–50%), 4 = greater than half of neurons damaged (51–75%), 5 = majority or all of neurons damaged, including infarction (>75%) [75,76]. 

### 4.5. Immunohistochemistry

Immunohistochemistry was performed on paraffin embedded sections at 6 h and 48 h following hypoxia-ischemia (n = 3/group for each time point). Following deparaffinization, sections were hydrated in xylene and graded alcohol series. For antigen retrieval, sections were treated with citrate buffer (pH = 6.0, for 1 h). Next, sections were treated overnight (4 °C) with primary monoclonal antibody against iNOS (sc-7271, Santa Cruz Biotechnology, Dallas, TX, USA, 1:100). The following day, a goat anti-mouse secondary antibody was applied for 1 h at room temperature (Dako, Agilent Technologies, Santa Clara, CA, USA, 1:200). In order to visualize immunoreactions, we used an avidin–biotin peroxidase complex (Vecstatin Kit; Vector Laboratories, Newark, CA, USA) with 3,3′-diaminobenzidine (DAB; Vector Laboratories, Newark, CA, USA) as the chromogen. All sections were dehydrated and counterstained with hematoxylin. As negative control, sections without primary antibody were used.

### 4.6. Statistical Analysis

All statistical analyses were created using the SPSS Statistics software (version 25). One-way repeated-measures ANOVA was used for analyzing numeric data on the Rota-Rod, grip traction and foot fault tests (groups: SHAM, HI/SAL and HI/NAC). Post-hoc comparisons using Bonferroni correction were conducted when necessary. The Cochran’s Q test was used to analyze the dichotomous variables of postural reflex and limb placing tests in each group. If the difference was statistically significant, the McNemar test was used for pairwise comparisons. Chi squared test was then applied for group comparison per day. Chi squared was also used to evaluate the grades of brain damage. Results were expressed as means ±SD and differences were considered statistically significant when *p* < 0.05.

## Data Availability

Not applicable.

## Supplementary Materials

The following supporting information can be downloaded at: https://www.mdpi.com/article/10.3390/ijms232416175/s1.
