# Supplementary material for: N-Acetylcysteine Administration Attenuates Sensorimotor Impairments Following Neonatal Hypoxic-Ischemic Brain Injury in Rats
Source: Int J Mol Sci. 2022 Dec 19;23(24):16175. doi: 10.3390/ijms232416175 (PMC9783020; doi:10.3390/ijms232416175)
Supplement: Supplementary file 1 [file ijms-23-16175-s001.zip › ijms-2045973-supplementary.pdf]

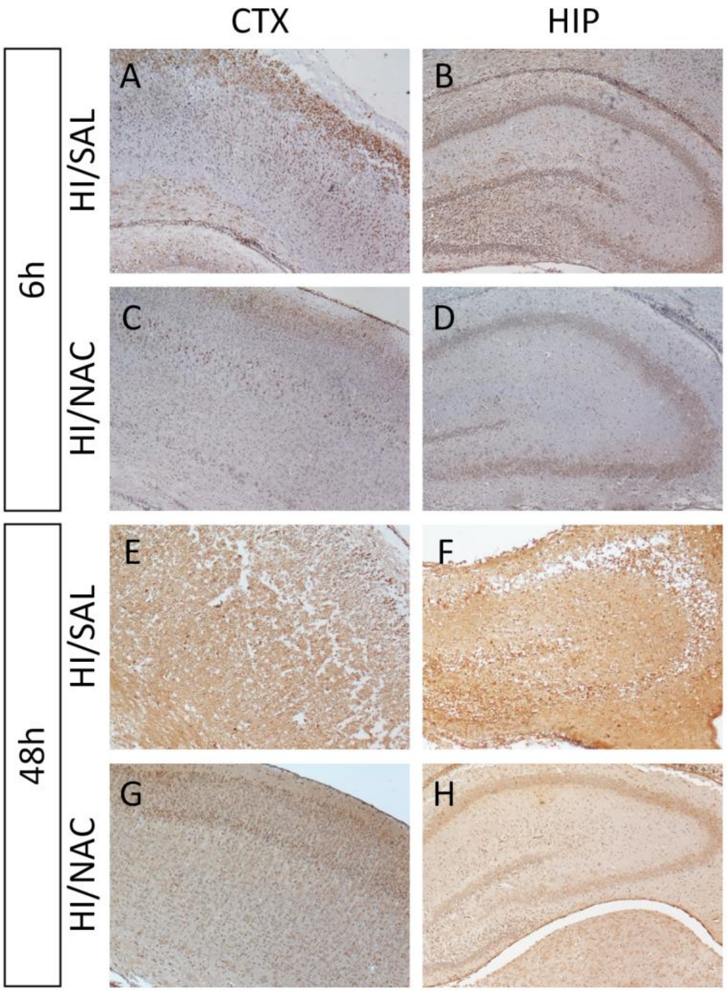

Figure S1. Brain images from which the representative photos were used in Figure 5 in the manuscript. Immunohistochemical staining for iNOS expression (DAB-brown staining) in the cortex-CTX (A,C,E,G) and hippocampus-HIP (B, D, F, H) from each group at 6h and 48h following neonatal HI. Lower levels of iNOS expression are observed both in CTX and HIP in NAC/HI group compared to the HI/SAL. Regions with extensive damage appeared to have diminished iNOS expression due to neuronal loss.
